# Induction of autophagy by sphingosine kinase 1 inhibitor PF-543 in head and neck squamous cell carcinoma cells

**DOI:** 10.1038/cddiscovery.2017.47

**Published:** 2017-08-14

**Authors:** Masakazu Hamada, Hiroyasu Kameyama, Soichi Iwai, Yoshiaki Yura

**Affiliations:** 1 Department of Oral and Maxillofacial Surgery, Osaka University Graduate School of Dentistry, Osaka, Japan

## Abstract

Sphingosine kinase 1 (SphK1) overexpressed in head and neck squamous cell carcinoma (SCC) regulates tumor growth. The effects of PF-543, a specific SphK1 inhibitor, on human SCC cells were examined. The proportion of viable cells after PF-543 treatment decreased in a time- and dose-dependent manner, and cell death occurred in SphK1-expressing SCC cells. Flow cytometry analysis revealed that PF-543 induced both necrosis and apoptosis. PF-543 also induced granular accumulation of LC3 and conversion from LC3-I to LC3-II, which was blocked by autophagy inhibitors, wortmannin, 3-methyladenine (3-MA), and bafilomycin A1. Treatment of head and neck SCC cells with autophagy inhibitors and PF-543 increased the proportion of cells with necrosis and apoptosis, indicating that autophagy acts to promote cell survival. Reactive oxygen species (ROS) scavenger reduced the cytotoxicity of PF-543. These results demonstrated that PF-543 induces apoptosis, necrosis, and autophagy in human head and neck SCC cells, and that autophagy antagonizes either necrosis or apoptosis.

## Introduction

Sphingolipids, especially ceramide, sphingosine, and sphingosine-1-phosphate (S1P) have an important role as regulatory molecules of cancer development. Although S1P promotes cell proliferation and survival, and regulates angiogenesis, sphingosine and ceramide inhibit cell proliferation and stimulate apoptosis. The S1P product is important in the regulation of a variety of biological processes, including Ca^2+^ mobilization, cytoskeleton rearrangement, cell proliferation, differentiation, survival and motility through activity as an intracellular second messenger and an extracellular ligand for G protein coupled receptors.^1–3^

S1P is formed through phosphorylation of sphingosine in intracellular compartments by sphingosine kinases (SphKs).^3–5^ In human cells, two isozymes, SphK1 and SphK2, are known. SphK1 has been found to be overexpressed in many types of human cancers including prostate cancer, gastric cancer, breast cancer, lung cancer, glioma, Hodgkin's lymphoma, and head and neck SCC.^3–5^ It is involved in tumor progression, invasion, metastasis, and radiation and chemoresistance.^3–5^ In head and neck SCC, elevated SphK1 levels are associated with poor outcomes and a reduction in SphK1 levels is associated with increased patient survival.^3^ Therefore, SphK1 is believed to be a promising target for cancer and inflammatory diseases.

The first known SphK inhibitors were N, N-dimethyl-d-erythro-sphingosine (DMS) and l-threo-dihydro-sphingosine (safingol).^6–9^ DMS inhibits both SphK1 and SphK2 by competing with natural substrates. Safingol is a saturated analog of sphingosine and is a protein kinase C (PKC) inhibitor with SphK inhibitory properties.^10^ In combination with cisplatin, safingol has been successfully tested in phase I clinical trials of advanced solid tumors.^6^ Another compound, 2-(p-hydroxyanilino)-4-(p-chlorophenyl) thiazole (SKI-II), is widely used as a SphK1 and SphK2 inhibitor.^11^ The sphingosine analog FTY 720 is a drug that demonstrates great potential for kidney transplantation and the management of chronic autoimmune diseases such as multiple sclerosis. FTY 720 is phosphorylated by SphK1 and the phosphorylated compound is a potent agonist of all S1P receptors (S1PR) except S1P2.^12^ (R)-[1-(4-{[3-methyl-5-(phenylsulfonylmethyl) phenoxy] methyl} benzyl) pyrrolidin-2-yl] methanol (PF-543) is a novel SphK1 inhibitor reported in 2012 and has 100-fold greater selectivity for SphK1 compared with SphK2.^13^

Autophagy is a catabolic process in which cytoplasmic components are sequestered in membrane-enclosed autophagosomes and delivered to lysosomes for degradation. Autophagy begins with the isolation of a double membrane bound structure. These membrane structures are elongated and microtubule-associated protein 1 light chain 3 (LC3) is recruited to the membrane.^14–16^ Elongated double membrane forms autophagosomes and sequester cytoplasmic proteins and organelles. After that, the autophagosomes mature, fuse with lysosomes, and become autolysosomes. Subsequently, the isolated contents are digested with lysosomal hydrolase and recycled. Decomposition by autophagy is generally thought to be a cytoprotective mechanism that maintains homeostasis in case of nutrient deficiency or exposure to environmental stress such as hypoxia. Paradoxically, several studies have shown that induction of autophagy can also contribute to caspase-dependent or independent programmed cell death.^17,18^

A number of anti-neoplastic therapies, including radiation therapy, chemotherapy, histone deacetylase inhibitors, arsenic trioxide, TNF-*α*, IFN-*γ*, imatinib and rapamycin, have been demonstrated to reduce autophagy as a protective or pro-survival mechanism in human cancer cell lines. Indeed, if autophagy is inhibited, the therapeutic effects of these drugs can be enhanced.^17^ Recent studies reported that the novel SphK1 inhibitor PF-543 exhibited potent anti-proliferative and cytotoxic effects on human colorectal cancer cells.^19^ It mainly induced programmed necrosis, but did not induce apoptosis. However, the involvement of autophagy in PF-543-induced cell death remains unclear. In the present study, we examined PF-543-induced autophagy in human head and neck SCC cells, and the role in several cell death mechanisms using autophagy inhibitors.

## Results

### Expression of SphK1 in head and neck SCC cells

**Figure 1:**
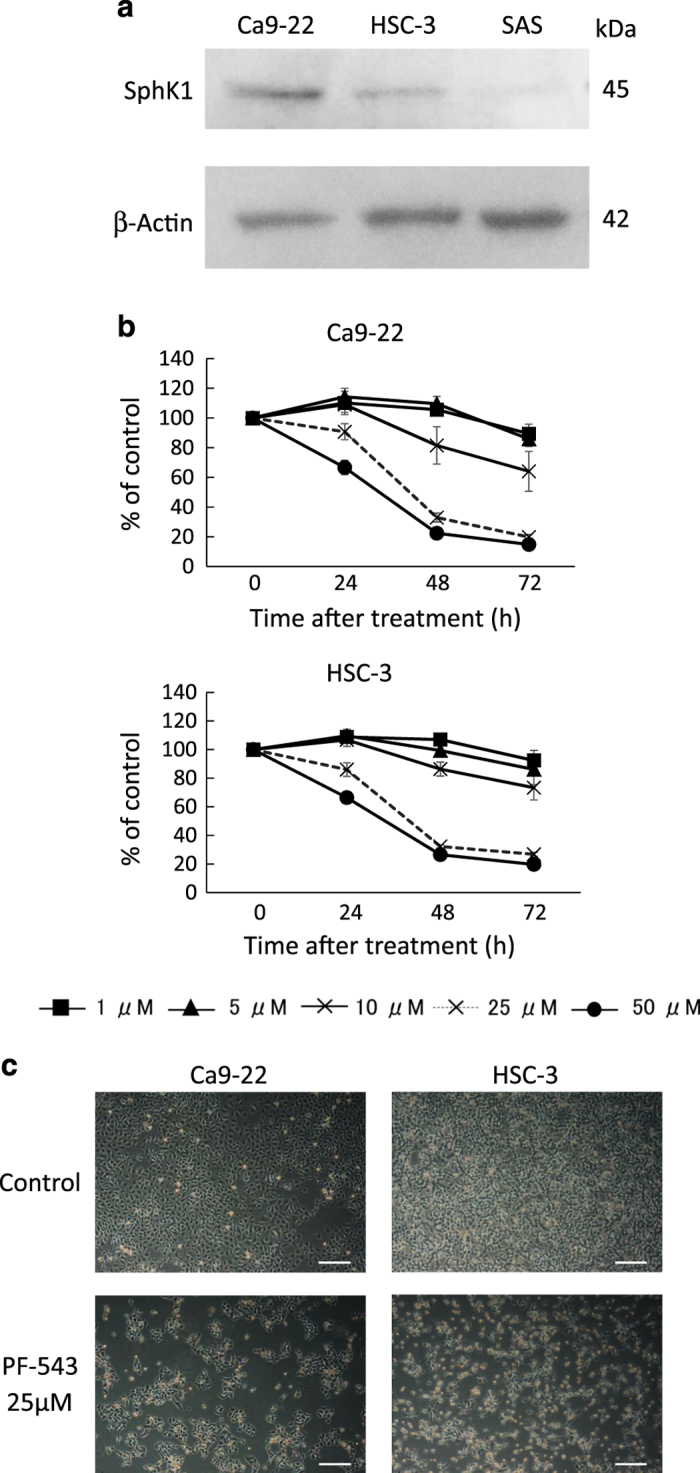
Inhibition of cell viability by PF-543. (**a**) The expression of SphK1 in Ca9-22, HSC-3 and SAS cells was examined by immunoblotting. (**b**) Cells (2.5×10^3^) in 96-well culture plates were cultured for 24 h before experiments. Ca9-22 and HSC-3 cells were treated with several concentrations of PF-543 for 24, 48 and 72 h, and cell viability was measured by MTT assay. Data are mean±S.D. (*n*=3). (**c**) Ca9-22 and HSC-3 cells were treated with PF-543 at a concentration of 25 *μ*M for 72 h and the cell morphology was photographed with a phase contrast microscope. Scale bar: 200  *μ*m.

To determine the expression of SphK1, head and neck SCC cell lines, Ca9-22, HSC-3 and SAS cells were subjected to immunoblotting using antibodies against SphK1. Immunoblot analysis revealed that expression of SphK1 protein was observed in all SCC cells at different levels (Figure 1a). Expression of SphK1 in SAS cells was lower compared with other cell lines.

### Inhibition of cell survival by PF-543

The effects of PF-543 on SCC cell viability were examined by MTT assay. In this study, Ca9-22 and HSC-3 cells with clearly detected SphK1 were used, and cells were cultured in the presence of PF-543 at concentrations of 1, 5, 10, 25 or 50 *μ*M for 24, 48 and 72 h. As a result, PF-543 dose- and time-dependently inhibited cell proliferation and the cell viability of Ca9-22 cells treated with 25 *μ*M PF-543 was 19.8% of the untreated control (Figure 1b). When HSC-3 cells were treated with 25 *μ*M PF-543 for 72 h, the proportion of viable cells decreased to 26.7% of the control (Figure 1b). The morphology of these SCC cells was affected by 25 *μ*M PF-543. The cells rounded and bound to adjacent cells in a fine intercellular cell process in 24 h. This morphological change was maintained at 72 h (Figure 1c).

### Cell death induction by PF-543

**Figure 2:**
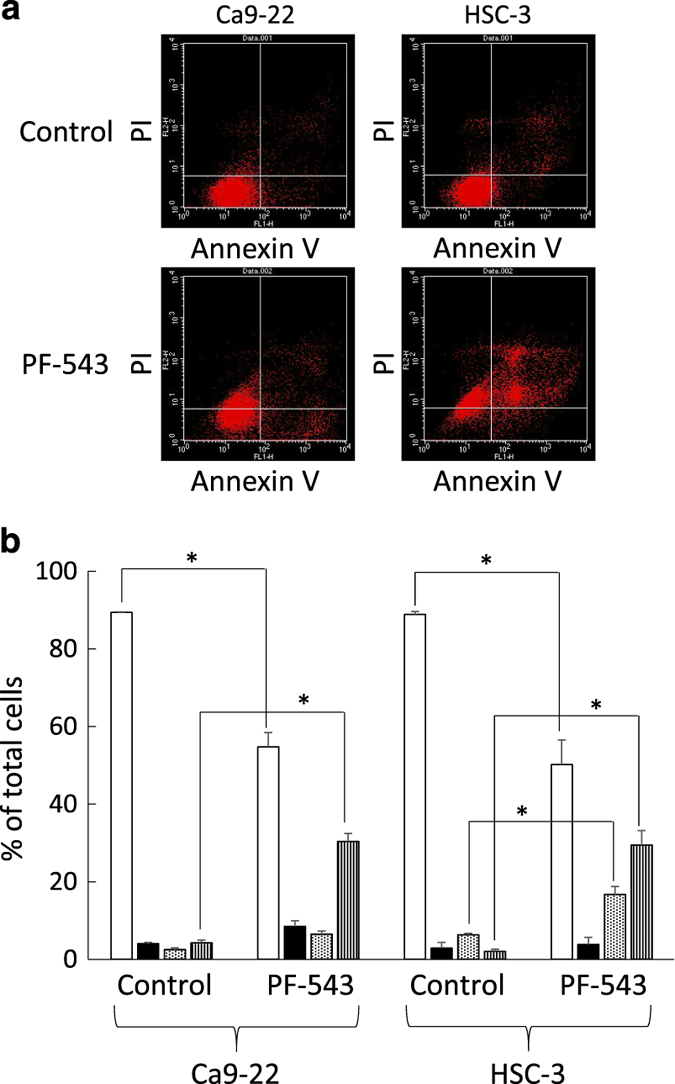
Induction of cell death by PF-543. (**a**) Cells (1.0×10^5^) in 6-well culture plates were cultured for 24 h before experiments. Ca9-22 and HSC-3 cells were treated with 25 *μ*M PF-543 for 72 h, stained with FITC-annexin V and PI, and subjected to flow cytometry. (**b**) Percentage of annexin V-negative and PI-negative viable cells (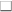), annexin V- positive and PI-negative early apoptotic cells (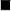), annexin V-positive and PI-positive late apoptotic cells (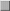), and annexin V-negative and PI-positive necrotic cells (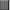) were determined. Data are mean±S.D. (*n*=3). *P<0.05, unpaired two-tail *t*-test.

Ca9-22 and HSC-3 cells were treated with 25 *μ*M PF-543 for 72 h, stained with annexin V-conjugated to fluorescein isothiocyanate (FITC-annexin V) and propidium iodide (PI), and subjected to flow cytometry (Figure 2a). The cell population was classified into 4 types, Annexin V-negative and PI-negative viable cells, Annexin V-positive and PI-negative early apoptotic cells, Annexin V-positive and PI-positive late apoptotic cells, and Annexin V-negative and PI-positive necrotic cells. The proportion of viable cells in Ca9-22 cells was 54.7%, and when treated with PF-543, the proportion of necrotic cells increased to 30.4% (Figure 2b). In the case of HSC-3 cells, the proportion of viable cells, necrotic cells and late apoptotic cells were 50.1%, 29.4% and 16.7%, respectively (Figure 2b).

### Induction of autophagy by PF-543

**Figure 3:**
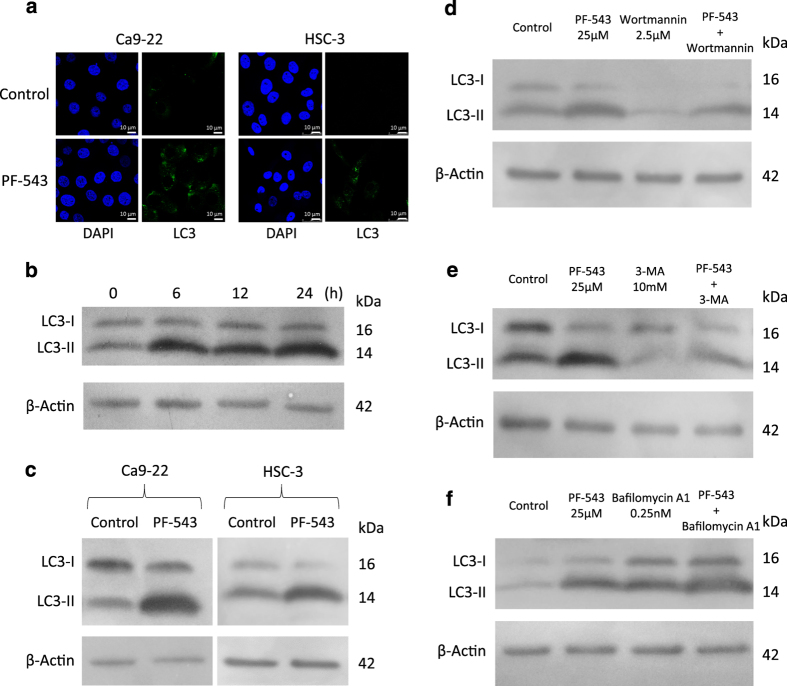
Induction of autophagy by PF-543. (**a**) Cells (1.0×10^5^) in 6-well culture plates were cultured for 24 h before experiments. Ca9-22 and HSC-3 cells were treated with 25 *μ*M PF-543 for 72 h and the expression of LC3 was examined by immunofluorescent antibody staining with anti-LC3 antibody and DAPI. (**b**) Expression of LC3-I and LC3-II in HSC-3 cells treated with PF-543 was examined for 24 h by immunoblotting. (**c**) Expression of LC3-I and LC3-II in Ca9-22 and HSC-3 cells treated for 72 h with PF-543 was examined by immunoblotting. HSC-3 cells were treated with 25 *μ*M PF-543 in combination with 2.5 *μ*M wortmannin (**d**), 10 mM 3-MA (**e**), or 0.25 nM bafilomycin A1 (**f**) for 72 h, and LC3-I and LC3-II were detected by immunoblotting.

Immunofluorescence staining was performed using anti-LC3 antibody and 4′,6-diamidino-2-phenylindole (DAPI) to determine the localization of LC3, the main component of autophagosomes. In untreated Ca9-22 and HSC-3 cells, a weakly diffuse cytoplasmic stained image was observed. On treatment of Ca9-22 and HSC-3 cells with 25 *μ*M PF-543 for 72 h, a granular pattern due to strong accumulation of LC3 was observed in the cytoplasm (Figure 3a).

Upon induction of autophagy, LC3-I changes to LC3-II and accumulates in autophagosomes formed in the cytoplasm.^16^ On immunoblot analysis of HSC-3 cells, conversion from LC3-I to LC3-II occurred 6 h after the start of PF-543 treatment and its level was maintained after 24 h (Figure 3b). LC3-II levels of Ca9-22 and HSC-3 cells treated with PF-543 were higher than those in untreated controls (Figure 3c).

### Enhancement of cell death by autophagy inhibitors

Wortmannin and 3-methyladenine (3-MA) are known inhibitors of autophagosome formation, and bafilomycin A1 inhibits the formation of autophagosomes and autolysosomes.^20–22^ Immunoblot analysis of LC3 after treatment of HSC-3 cells with PF-543 in combination with these autophagy inhibitors demonstrated that wortmannin and 3-MA reduced LC3-II expression levels (Figures 3d and e). In cells treated with bafilomycin A1, high LC3-II levels were maintained (Figure 3f).

**Figure 4:**
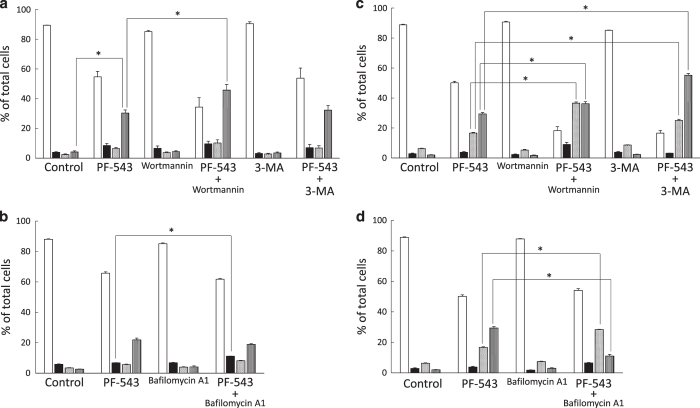
Enhancement of cell death by autophagy inhibitor. Cells (1.0×10^5^) in 6-well culture plates were cultured for 24 h before experiments. Ca9-22 cells were treated for 72 h with PF-543 and wortmannin (**a**), 3-MA (**a**), or bafilomycin A1 (**b**), stained with FITC-annexin V and PI, and subjected to flow cytometry. HSC-3 cells were treated with PF-543 in combination with wortmannin (**c**), 3-MA (**c**), or bafilomycin A1 (**d**). The percentage of viable cells (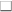), early apoptotic cells (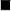), late apoptotic cells (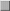), and necrotic cells (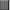) was determined. Data are mean±S.D. (*n*=3). *P<0.05, unpaired two-tail *t*-test.

To know the effects of PF-543 on the mode of cell death, Ca9-22 and HSC-3 cells were treated with PF-543, and the populations of viable cells, apoptotic cells and necrotic cells were analyzed with a flow cytometer. Wortmannin, 3-MA, and bafilomycin A1 alone exhibited no significant inhibitory effects on the proportion of viable cells at the working concentration. The proportion of necrotic cells after treatment of Ca9-22 cells for 72 h with PF-543 alone was 30.4%, but it increased to 45.8% with PF-543 and wortmannin (Figure 4a). Treatment with PF-543 and bafilomycin A1 resulted in a slight increase in early apoptotic cells (Figure 4b). In HSC-3 cells, after treatment with PF-543 in combination with wortmannin or 3-MA, the proportions of necrotic cells were 36.2 and 55.2% respectively, indicating that autophagy inhibitors further increased necrotic cells. A significant difference (*P*<0.05) was observed between PF-543 alone and PF-543 with wortmannin or 3-MA (Figure 4c). When HSC-3 cells were treated with PF-543 in combination with bafilomycin A1, late apoptotic cells increased from 16.7 to 28.4%. In contrast, the proportion of necrotic cells decreased from 29.4 to 11.1% (Figure 4d).

### Suppression of cell death by the ROS scavenger NAC

**Figure 5:**
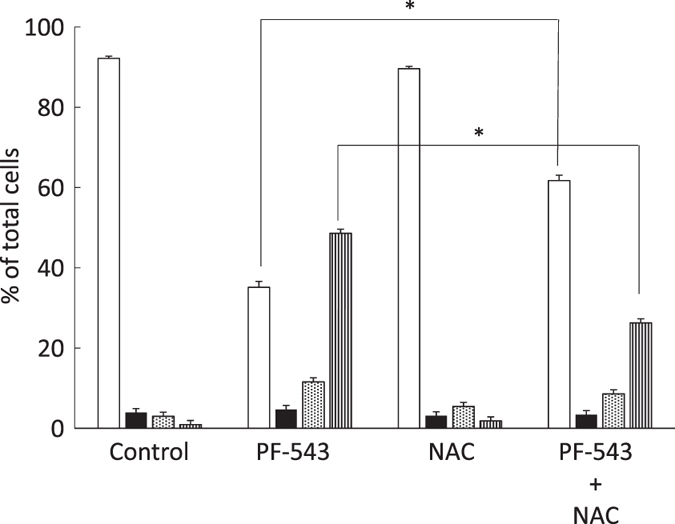
Suppression of cell death by the ROS scavenger, NAC. Cells (1.0 x 10^5^) in 6-well culture plates were cultured for 24 h before experiments. HSC-3 cells were pretreated with NAC for 2 h, then treated with PF-543 in the presence of NAC for 72 h. They were stained with FITC-annexin V and PI, and then subjected to flow cytometry. The percentage of viable cells (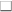), early apoptotic cells (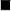), late apoptotic cells (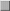) and necrotic cells (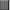) was determined. Data are mean±S.D. (*n*=3). *P<0.05, unpaired two-tail *t*-test.

Bioactive lipids, such as ceramides, have been reported to induce reactive oxygen species (ROS) production.^23–25^ The effect of a ROS scavenger, N-acetyl-l-cysteine (NAC), on PF-543 induced cell death was investigated. HSC-3 cells were pretreated with NAC and treated with PF-543 for 72 h, then analyzed using flow cytometry. The proportion of necrotic cells induced by PF-543 decreased from 48.6 to 26.3%. There was a significant difference between these groups (Figure 5), indicating an important role of ROS in cell death.

## Discussion

SphK1 is overexpressed in head and neck SCC, and positively correlates with invasion and reduced sensitivity to radiotherapy.^3–5^ We have demonstrated that safingol induces apoptosis and autophagy in head and neck SCC cells.^26–28^ Recent studies reported anti-proliferative and cytotoxic effects of PF-543 on a panel of established and primary human colorectal cancer cells.^19^ Based on these findings, we decided to investigate the possibility that PF-543 induced cell death and autophagy in head and neck SCC cells.

First, the expression of SphK1 in SCC cell lines derived from head and neck SCC was examined by immunoblot analysis, and expression of SphK1 was found in all tested cell lines, but its expression was diverse. Testing the ability to inhibit proliferation of SCC cells using Ca9-22 and HSC-3 cells expressing SphK1 at high levels demonstrated that cell proliferation decreased in a time- and dose-dependent manner. Morphological changes, mainly cell rounding, appeared 48 h after incubation with PF-543. In the first report, PF-543 did not exert strong inhibitory effects at low concentration on 1483 cells.^13^ However, after incubation for 4 days at a concentration of 10 *μ*M, significant inhibition of proliferation of colorectal cancer cells was reported.^19^ As PF-543 inhibits the activity of SphK1 and reduces the level of S1P, which is essential for suppressing cell proliferation, long-term treatment may be necessary to exert anti-proliferative effects. When incubated for 48 h or more at a concentration of 25 *μ*M, PF-543 appears to exhibit cytotoxic effects on head and neck SCC cells.

PF-543 was also found to induce cell death in head and neck SCC cells by flow cytometry analysis. In this analysis, the type of cell death was classified as viable cells, early apoptotic cells, late apoptotic cells or necrotic cells. The proportion of viable cells was reduced when SCC cells were treated with PF-543 for 72 h, and necrosis was a major cause of cell death.

Consistent with the results of this study, Ju *et al.*^19^ reported that PF-543 primarily induced necrotic cell death in human colorectal cancer cells, but they did not examine the involvement of autophagy. Recently, we discovered autophagy as well as apoptosis occurred at concentrations of safingol that induce morphological changes in SCC cells.^28^ In the present study, we found a granular pattern of LC3 exhibiting autophagosome formation and the conversion of LC3-I to LC3-II in PF-543-treated Ca9-22 and HSC-3 cells. Therefore, we concluded that PF-543 induced autophagy after treatment at concentrations that had cytotoxic effects.

Although autophagy induction by PF-543 has not been reported to date, other SphK inhibitors, the SphK1/SphK2 inhibitor SKI-II and the SphK2 inhibitor ABC 294640, have been shown to induce autophagy.^29,30^ McNaughton *et al.*^31^ reported that SKI-II and ABC 294640 inhibited DNA synthesis in androgen-independent LNCaP-AI prostate cancer cells, inducing proteosomal degradation of SphK1. These effects were reproduced by inhibitors of dihydroceramide desaturase (Des 1), which is the last enzyme in the novel synthesis of ceramide to convert dihydroceramide to ceramide. As dihydroceramide accumulated by blocking Des 1, a known inducer of autophagy,^32^ growth arrest induced by SKI-II or ABC 294640 is considered to be mediated by SphK1 and Des 1.^31^ Therefore, PF-543 may demonstrate cytotoxicity by increasing the amounts of phospholipid metabolites, which induce autophagy.

Recent studies suggest that factors in the apoptotic pathway also exert regulatory activity on factors in autophagy.^33–35^ For example, anti-apoptotic Bcl-2 family proteins that downregulate apoptosis by antagonizing the activity of pro-apoptotic proteins can downregulate autophagy. Beclin 1 interacts with anti-apoptotic Bcl-2 family members, including Bcl-2 and Bcl-xL. Binding of these Bcl-2 family proteins to Beclin 1 inhibits autophagy by altering the association of Beclin 1 and Class III phosphatidylinositol 3-kinase (PI3K) complexes.^36^ Ding *et al.*^29^ reported that ABC 294640-induced apoptosis in cholangiocarcinoma cells was enhanced when autophagy was inhibited by bafilomycin A1 or chloroquine. We showed that safingol induced endonuclease G-dependent apoptosis and autophagy of head and neck SCC cells, and this induced autophagy acted as a survival promoter.^28^ On the other hand, Goodall *et al.*^37^ reported that the deficiency of tumor suppressor gene MAP3K7 in mouse prostate cells increased sensitivity to cell death by TNF-related apoptosis-inducing ligand (TRAIL). This cell death occurred primarily through necrosomes and was not apoptosis due to assembly of necrosomes in relation to the autophagy mechanism. Necrosis cell death became apoptosis if the p62-dependent mobilization of necrosomes to the autophagy mechanism was prevented. These earlier findings suggest that autophagy affects whether cells will die, as well as controls other aspects of programmed cell death such as apoptosis and necrosis.

**Figure 6:**
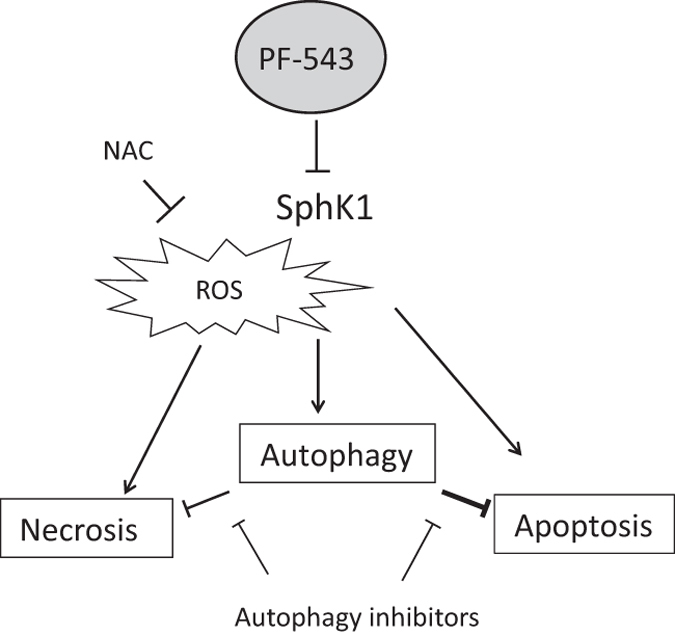
Schematic representation of the effects of PF-543 on head and neck SCC cells. PF-543 inhibits the activity of SphK1 in head and neck SCC cells, decreases S1P and induces necrosis and apoptotic cell death, as well as autophagy. Autophagy inhibitors can block the inhibitory effects of autophagy on necrosis and apoptotic pathways, and can enhance PF-543-induced cytotoxicity. ROS is a mediator of cell death induced by PF-543.

In the present study, we found that autophagy inhibitors, wortmannin, 3-MA and bafilomycin A1 efficiently prevented the processes of autophagy. We also confirmed that these autophagy inhibitors alone do not affect the cell viability of head and neck SCC cells. Then, flow cytometry analysis was performed by treating Ca9-22 and HSC-3 cells with PF-543 and wortmannin or 3-MA, and as a result, necrotic cells significantly increased in comparison with PF-543 alone, but early and late apoptotic cells were not significantly affected. This increase in necrotic cells by inhibiting autophagy was thought to enhance the cytotoxicity of PF-543 on head and neck SCC cells. Thus, autophagy induced by PF-543 can act to prevent necrotic cell death (Figure 6). ‘Cross-talk’ of autophagy and necrosis^38^ may be related with an increase in the necrotic cell population due to inhibition of autophagy by wortmannin and 3-MA. In the case of bafilomycin A1, the change in necrosis was minimal and the apoptotic cells increased. Cell death patterns of Ca9-22 and HSC-3 cells may be switched from necrosis to apoptosis by inhibition of autophagy with bafilomycin A1.

Necrosis mediators are excess cytoplasmic Ca^2+^ and levels of ROS.^39^ Previous studies have demonstrated that ROS production can mediate apoptosis and/or autophagy induction in several types of cancer cells.^23^ Ling *et al.*^24^ reported an important role of ROS in necrosis of safingol-treated cells. We also reported ROS production by safingol in head and neck SCC cells.^25^ In this study, we found that the removal of ROS by N-acetyl-L-cysteine (NAC) prevented the production of necrotic cells by PF-543. Therefore, it is likely that ROS generated by PF-543 is a mediator of PF-543-induced cell death and autophagy.

In conclusion, our results demonstrated that PF-543 induced apoptosis, necrosis and autophagy in human head and neck SCC cells, and that induced autophagy antagonized both necrosis and apoptosis.

## Materials and methods

### Cells

The human oral SCC cell line SAS and HSC-3 cells were obtained from the RIKEN BRC CELL BANK (Tsukuba, Japan), and Ca9-22 cells were from the Japanese Collection of Research Bioresources (Tokyo, Japan). Cells were cultured in Dulbecco’s modified Eagle’s medium supplemented with 5% fetal bovine serum, 4 mM l-glutamine, 100 *μ*g/ml penicillin and 100 *μ*g/ml streptomycin, and then grown in an incubator at 37 °C in a humidified atmosphere with 5% CO_2_.

### Reagents

Sphingosine kinase 1 inhibitor (PF-543) was obtained from Merck KGaA (Darmstadt, Germany) and a stock solution was made in dimetyl sulphoxide (DMSO). Wortmannin, 3-MA, bafilomycin A1, and MTT were obtained from Sigma (St. Louis, MO, USA). NAC was obtained from Wako (Osaka, Japan).

### MTT assay

Cells grown in 96-well culture plates were treated with PF-543. Thereafter, 10 *μ*l of 5 mg/ml MTT solution was added to each well with 100 *μ*l of medium and cells were incubated at 37 °C for 4 h. After the addition of 100 *μ*l of 0.04 N HCl in isopropanol, the plates were mixed thoroughly to dissolve the dark blue crystals and remained at room temperature overnight. The plates were read on a Benchmark Plus microplate spectrophotometer (Bio-Rad Laboratories, Hercules, CA, USA) with a reference wavelength of 630 nm and a test wavelength of 570 nm. Background absorbance at 630 nm was subtracted from the 570 nm reading.

### Flow cytometry analysis

FITC-annexin V and PI staining was performed using Vybrant Apoptosis Assay Kit #3 (Life Technologies, Carlsbad, CA, USA) following the manufacturer’s directions. After being treated, floating cells were harvested with medium and attached cells were dissociated with EDTA-trypsin solution. Cells were collected by centrifugation at 1000 r.p.m. for 5 min. Cells were centrifuged and washed twice with phosphate-buffered saline (PBS), and the pellets were suspended in 100 *μ*l binding buffer containing 10 mM HEPES, 140 mM NaCl and 2.5 mM CaCl (pH 7.4), and incubated with 5 *μ*l of FITC-annexin V and 1 *μ*l of 100 *μ*g/ml PI solution for 15 min at room temperature. Thereafter, 400 *μ*l of binding buffer was added, mixed gently, and kept on ice. Stained cells were analyzed by FACSCalibur (Becton Dickinson, Mountain View, CA, USA). Data were analyzed by Cell Quest software (Becton Dickinson).

### Immunoblot analysis

To detect proteins, cells were washed in PBS and lysed in a buffer containing 20 mM Tris-HCl (pH 7.4), 0.1% SDS, 1% Triton X-100, 1% sodium deoxycholate and protease inhibitor cocktail. After sonication on ice and subsequent centrifugation at 15 000×*g* for 10 min at 4 °C, the supernatant was collected and the protein concentration was determined using a Protein Assay Kit (Bio-Rad, Hercules, CA, USA). A protein sample (20 *μ*g) was electrophoresed through a polyacrylamide gel and transferred to a PVDF membrane (Millipore, Bedford, MA, USA) by electroblotting. The membrane was probed with antibodies and antibody binding was detected using an enhanced chemiluminescence (ECL) kit (GE Healthcare, Amersham, Buckinghamshire, UK) according to the manufacturer’s instructions. The antibodies used were as follows: rabbit polyclonal antibodies against SphK1 (Cell Signaling Technology, Beverly, MA, USA), mouse monoclonal antibodies against LC3 and *β*-actin (Medical & Biological Laboratories, Nagoya, Japan), and horseradish peroxidase-conjugated secondary antibodies (Cell Signaling Technology).

### Confocal laser microscopic analysis

After being treated, cells were fixed in 4% paraformaldehyde phosphate buffer solution (Wako) and incubated with an antibody against LC3 diluted 1:500 for 1 h at room temperature. After washing, the cells were incubated with an Alexa Fluor 488 goat anti-mouse IgG antibody (Life Technologies, Carlsbad, CA, USA) diluted 1:500 in PBS for 1 h. After washing, coverslips were mounted onto microslides using a ProLong Gold Antifade Reagent with DAPI (Life Technologies Corporation). The slides were analyzed with the confocal laser-scanning microscope Leica TCS SP8 (Leica Microsystems, Mannheim, Germany).

### Statistical analyses

Statistical analyses were performed using the Student’s *t*-test with Microsoft Excel (Microsoft, Redmond, WA, USA). Results were expressed as the mean±S.D. Differences were considered significant at *P*<0.05.

## Publisher’s note

Springer Nature remains neutral with regard to jurisdictional claims in published maps and institutional affiliations.
